# Endometrial receptivity failure in assisted reproduction: immune-cellular niches, mechanistic evidence, and translational boundaries

**DOI:** 10.3389/fimmu.2026.1942809

**Published:** 2026-09-21

**Authors:** Yuhang Zhang, Yi Zhu, Zhipeng Li, Jing Luo

**Affiliations:** 1 Department of Reproductive Medicine, 116 Hospital, China RongTong Medical Healthcare Group Co. Ltd., Anqing, China; 2 Department of Central Laboratory, Binhai County People’s Hospital, Yancheng, China

**Keywords:** ART add-on interventions, chronic endometritis, embryo-endometrial interface, endometrial receptivity, microbiota, recurrent implantation failure, reproductive immunology, single-cell transcriptomics

## Abstract

Endometrial receptivity failure and recurrent implantation failure (RIF) are clinically heterogeneous outcomes shaped by embryo competence, uterine anatomy, endocrine support, immune adaptation, and the timing of embryo-endometrial synchrony. This Review focuses on how immune-cellular niches, cell-specific and spatiotemporal mechanisms, disease-modified receptivity states, and evidence-to-translation boundaries should be interpreted in assisted reproduction. Single-cell, spatial, and multi-omics studies increasingly resolve abnormalities in stromal, sepithelial, vascular, perivascular, and immune compartments, including altered decidualization, epithelial competence, inflammatory tone, metabolic signaling, and microbiota-immune communication. However, most of these signals remain mechanistic, preclinical, associative, or candidate-biomarker evidence rather than proof of clinical utility. Therefore, biological plausibility should not be conflated with routine care: transcriptome-based receptivity timing tests, empirical immune profiling or treatment, microbiota-directed interventions, endometrial scratching, and regenerative add-ons require cautious interpretation unless they improve clinically meaningful outcomes. Future progress depends on standardized RIF phenotyping, cycle-phase and sampling control, euploid embryo transfer where relevant, external validation of cell-specific markers, prospective trials, live-birth endpoints, safety follow-up, and transparent separation of discovery evidence from clinically actionable strategies.

## Graphical Abstract

**Figure:**
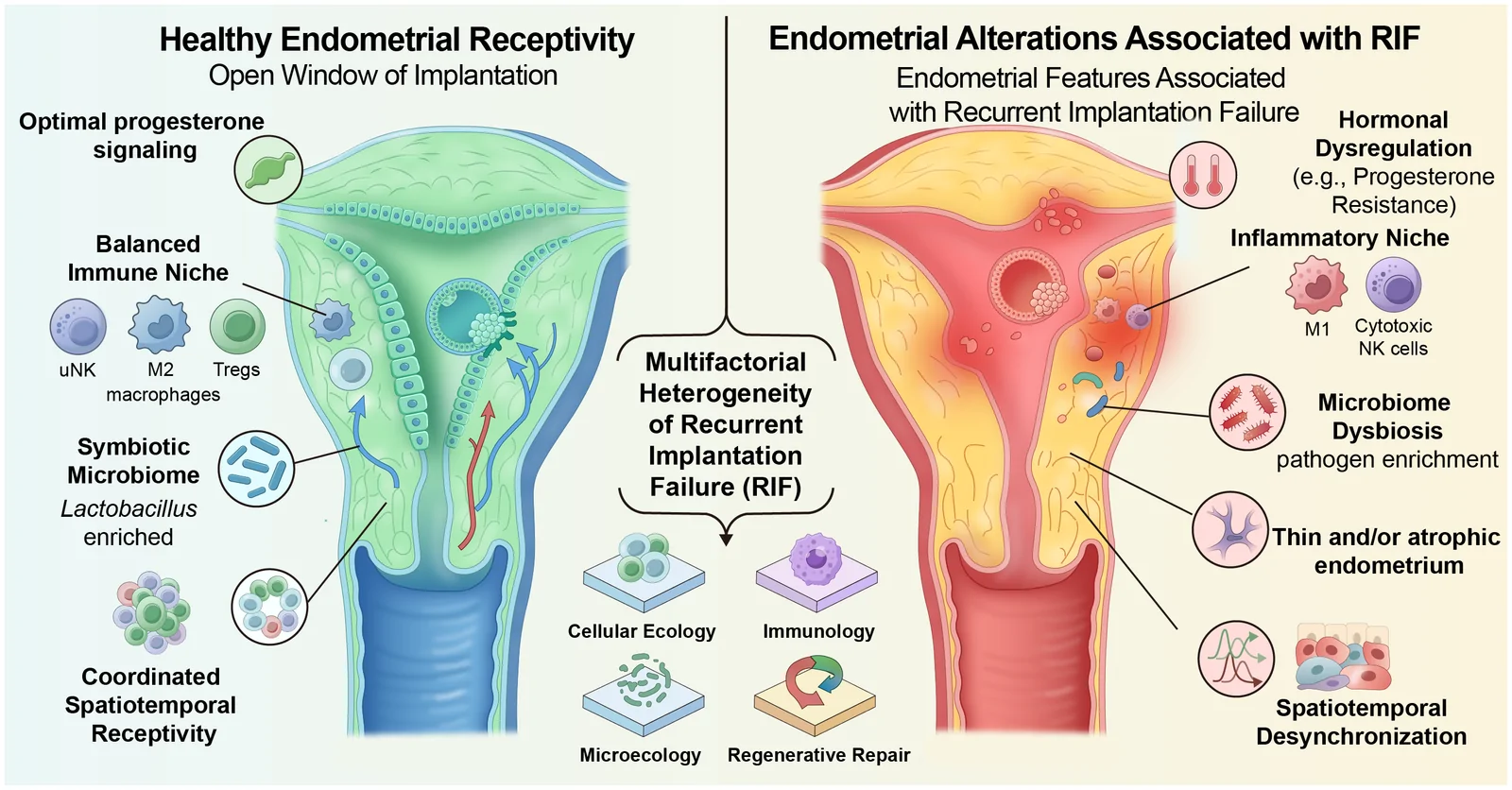
Multifactorial heterogeneity of recurrent implantation failure and abnormal endometrial receptivity. The graphical abstract summarizes the transition from a receptive endometrium to a non-receptive state associated with RIF. Successful implantation depends on progesterone responsiveness, balanced immune-cellular niches, a low-inflammation reproductive-tract microenvironment, and coordinated cellular and vascular remodeling. Abnormal receptivity may arise from hormonal dysregulation, inflammatory immune activation, microbiome dysbiosis, thin or atrophic endometrium, and spatiotemporal desynchronization.

## 1 Introduction

Embryo implantation is a coordinated biological process determined by embryonic developmental competence, chromosomal status, endometrial receptivity, local immune adaptation, endocrine support, uterine anatomy, and embryo-endometrial signaling (1, 2). Implantation failure should therefore not be attributed simplistically to a single endometrial abnormality. Organic lesions such as polyps, fibroids, adenomyosis, adhesions, or chronic inflammatory changes may be clinically relevant when they create a correctable phenotype, but their presence does not automatically establish causality or prove benefit from intervention (3–5). A useful clinical framework must first separate embryo-related probability, actionable uterine pathology, and endometrial functional hypotheses before considering empirical add-on treatment.

Endometrial receptivity is not a static molecular label. It is a transient, spatially organized tissue state generated by cyclic hormonal remodeling, stromal decidualization, epithelial adhesion and secretory maturation, vascular adaptation, immune-cell reorganization, and local microenvironmental signals (6, 7). Single-cell RNA sequencing, spatial transcriptomics, organoid models, and other omics platforms have improved mechanistic resolution by showing that bulk endometrial averages can mask region-specific and cell-type-specific abnormalities. Their main value at present is to refine biological hypotheses and disease phenotypes, not to replace clinical validation.

The clinical entity of RIF remains especially vulnerable to overdiagnosis because definitions, embryo status, transfer number, maternal age, and outcome measures vary across studies. Current evidence does not show that endometrial factors dominate all implantation failure, particularly in euploid embryo-transfer settings. The Lugano consensus cautioned that clinically meaningful RIF may be uncommon and recommended restraint before assigning the diagnosis, while Canadian clinical guidance similarly emphasizes individualized, evidence-based evaluation after correctable factors have been considered (8, 9). These perspectives make embryo/euploidy control and transparent phenotyping essential when interpreting endometrial mechanisms.

Accordingly, this Review asks how immune-cellular and microecological mechanisms of receptivity can be connected to clinically meaningful translation without overstating the evidence. The scope is not a catalog of biomarkers, a recommendation of ART add-ons, or proof that the endometrium is the primary cause of all RIF. Instead, it is a critical narrative synthesis linking stromal, epithelial, immune, vascular, metabolic, and microbiota-associated mechanisms with disease contexts and clinical-readiness boundaries.

We use an evidence-to-translation framework rather than a numerical evidence tier. Clinical efficacy evidence refers to randomized or appropriately controlled studies using reproductive outcomes, particularly ongoing pregnancy, live birth, and safety. Prospective association or prediction studies may support risk stratification without proving treatment benefit; retrospective and observational associations identify correlations; omics, single-cell, spatial, molecular, and machine-learning studies provide discovery or hypothesis-generating evidence; and *in vitro*, animal, organoid, or biomaterial studies provide preclinical mechanistic support. Animal models, organoids, discovery omics, and single-cell communication analysis are therefore not described as evidence of clinical efficacy. This framework structures the Review and is summarized in **Table 1**. These terms are used consistently throughout the text and **Tables 1**-**3**: ‘clinical efficacy’ is reserved for outcome-tested controlled human evidence, whereas observational findings are described as associations or predictions, and omics/model studies as discovery or preclinical mechanistic support.

**Table 1:** Mechanistic domains and evidence-to-translation boundaries in impaired endometrial receptivity.

Mechanistic domain	Core abnormality	Evidence domain/sources	Translational boundary	Clinical implication
WOI timing and maturation	Asynchronous endometrial state	Transcriptome-based timing studies and euploid-transfer systematic evidence (14–16, 139)	Classification of timing does not demonstrate outcome benefit.	Research or exceptional individualized use; not routine screening.
Stromal decidualization	Hormone response, cytoskeleton, fibrosis, senescence	Single-cell, spatial, molecular, *in vitro*, and animal data (18, 19, 21–26, 32)	Mechanistic/preclinical support is not clinical efficacy evidence.	Target discovery and phenotype refinement.
Luminal/glandular epithelium	Adhesion, polarity, secretory, and LIF-related changes	Single-cell, spatial, epithelial adhesion/polarity, LIF, and glandular-state studies (18, 19, 27–33)	Candidate epithelial states need replication across cycle phase and disease context.	Epithelial-state phenotyping for research stratification.
Immune and CE-associated inflammation	uNK, T-cell, macrophage, cytokine, CE, and microbiota shifts	uNK, T-cell, macrophage, cytokine, CE, and microbiota studies (63–88, 111–115, 140, 141)	High assay heterogeneity and nonstandardized CE thresholds.	Selective evaluation when clinically suspected; no empirical immunotherapy.
Disease-modified receptivity	Core disease contexts: endometriosis and PCOS; contextual modifiers: metabolic inflammation, obesity, and selected environmental/epigenetic factors	Endometriosis, PCOS, obesity, metabolic, exposure, and epigenetic evidence (41–62)	Disease association and model evidence do not define universal treatment algorithms.	Etiology-specific stratification and trial enrichment.
Embryo-endometrium communication	EVs, semen signals, lipid mediators, and microbiota-host signaling	EV/exosome, lipid, semen, and microbiota-host signaling evidence (89–97, 116–118)	Platforms, timing, contamination control, and functional validation remain incomplete.	Mechanistic and noninvasive biomarker research.
Repair failure and regeneration	Thin, aging, scarred, fibrotic, or senescent endometrium	Thin-endometrium single-cell evidence and regenerative/preclinical studies (11–13, 120–136, 142)	Thickness, angiogenesis, or animal fertility restoration is not live-birth benefit.	Regenerative approaches require standardized trials and safety follow-up.

The table organizes the principal biological domains discussed in the review and distinguishes clinical efficacy evidence, prospective or retrospective human association, discovery evidence, and preclinical mechanistic support.

**Table 2:** Diagnostic and assessment strategies for impaired endometrial receptivity and recurrent implantation failure.

Approach	Intended use	Evidence/representative sources	Main limitation	Clinical position
Ultrasound and morphology	Thickness, pattern, cavity, and perfusion assessment	Established clinical assessment and contextual evidence (4, 10–13)	Indirect surrogate for receptivity.	Routine baseline assessment.
Hysteroscopy/pathology for actionable lesions	Confirm and treat polyps, adhesions, hyperplasia, retained tissue, or suspicious CE	Clinical lesion-directed evidence (4, 5, 17, 76, 77)	Benefit depends on a confirmed actionable lesion.	Selective use when indicated.
Transcriptome-based receptivity timing tests (e.g., ERA)	WOI timing or maturation classification	Can classify transcriptomic timing, but controlled/euploid-transfer outcome benefit is unproven or inconsistent (14, 15, 139)	Prediction/classification is not clinical utility.	Not recommended for routine screening; research or exceptional individualized use.
Discovery transcriptomic/multi-omics profiling	Subtype, pathway, and candidate-biomarker discovery	Meta-analysis, spatial, single-cell, and public-dataset evidence (16, 18, 19, 38, 54)	Batch effects, overfitting, and external validation gaps.	Research setting.
Immune profiling	uNK cells, cytokines, T cells, macrophages, immune ratios	Systematic reviews and immune studies (63–72, 86–88)	Timing, source, cell definition, and assay heterogeneity.	Not routine as a stand-alone treatment guide.
CE confirmatory assessment (biopsy/CD138-supported histopathology)	Confirm plasma-cell-rich inflammatory phenotype when CE evaluation is clinically indicated.	Diagnostic heterogeneity and an association between CE and implantation failure are reported in clinical studies and evidence reviews (73–77, 140, 141).	Biopsy timing, CD138 staining protocol, and plasma-cell thresholds remain nonstandardized; hysteroscopy and microbiology are complementary.	Selective evaluation; not universal screening; antibiotics only for a confirmed infectious or inflammatory phenotype.
Microbiota testing	Vaginal/endometrial dysbiosis and host-response signatures	Sampling, culturomics, sequencing, and ART outcome studies (98–118)	Low biomass, contamination, and absence of a healthy-core definition.	Research or cautious selective use; no routine antibiotics/probiotics.
EV/lipidomic/metabolomic exploratory biomarkers	Noninvasive communication or metabolic signatures	EV and lipid-metabolism evidence (89–94)	No validated cutoffs or clinical-utility trials.	Research setting.

The table separates established assessment, selective lesion-directed evaluation, transcriptome-based receptivity timing tests, discovery omics, immune profiling, CE assessment, microbiota testing, and exploratory EV/lipidomic/metabolomic markers.

**Table 3:** Clinical-evidence and readiness matrix for therapeutic and ART add-on interventions.

Intervention	Mechanistic rationale	Clinical evidence/readiness	Main unresolved issue	Clinical position
Hormonal optimization	Synchronize estrogen/progesterone support and luteal phase	Established within ART protocols when clinically indicated (1, 2, 4)	Not a universal RIF solution.	Individualized clinical care.
GH/IGF-1	Possible ovarian and endometrial proliferative or signaling effects	Mechanistic and heterogeneous clinical evidence (120, 121)	Dose, timing, target group, and live-birth benefit unclear.	Investigational/selective.
PRP	Growth-factor-rich repair and angiogenic signaling	Low-certainty evidence; Cochrane PRP review uncertain (122–128)	Product standardization, endpoints, and safety.	Not routine.
G-CSF	Angiogenesis, immune, and repair signaling	Inconsistent small or observational evidence (129, 130)	Control groups and live-birth endpoints.	Not routine.
hCG/PBMC infusion	Embryo-endometrium immune signaling and cytokine modulation	Mixed low-certainty evidence (130, 131, 142)	Limited head-to-head RCTs and phenotype selection.	Research setting or selected use.
Endometrial scratching	Controlled injury hypothesized to induce repair/inflammatory signaling	Large RCT showed no live-birth benefit; 2026 Cochrane evidence remains uncertain (132, 133)	Pain, bleeding, heterogeneity, and lack of robust benefit.	Do not recommend routine empirical use.
Antibiotics for confirmed CE	Treat infectious/inflammatory phenotype	Supportive but heterogeneous evidence (73–80, 111–115, 140, 141)	Diagnostic threshold, recurrence, and antimicrobial stewardship.	Selective after confirmed diagnosis.
Probiotics/microbiota modulation	Modify reproductive tract ecosystem and immune tone	Associative or combined-intervention evidence (116, 118, 119)	Contamination control and live-birth evidence.	Research only.
Stem cells, exosomes, and biomaterials	Paracrine repair, angiogenesis, immune rebalancing, and scaffold-assisted regeneration	Preclinical and limited human evidence (131, 134–136)	Cell/EV characterization, dose, delivery, placentation, and long-term safety.	Research setting.
Lifestyle/metabolic care	Reduce systemic inflammation and metabolic risk	Supportive or observational evidence (55–59, 96, 97)	Causality, adherence, and disease stratification vary.	Supportive individualized care.

The table summarizes intervention categories and separates mechanistic rationale from clinically meaningful evidence, readiness, and unresolved safety or standardization issues.

### 1.1 Literature search and narrative evidence selection

This was a narrative review supported by a structured literature search; study selection supported critical synthesis rather than formal systematic review or meta-analysis. PubMed was searched on 5 September 2026 using the query: (endometrial receptivity) AND (recurrent implantation failure OR assisted reproduction OR IVF) AND (immune OR stromal OR decidualization OR epithelial OR microbiota OR chronic endometritis OR transcriptomics OR organoid OR single-cell OR spatial). The search retrieved 1,286 records. Literature selection for this narrative synthesis was guided by relevance to endometrial receptivity/RIF and to the mechanistic or clinical-translation scope of this Review. Targeted supplementary searches and citation chasing were used to incorporate additional high-relevance studies identified during revision. We prioritized human guidelines, randomized or controlled clinical evidence, systematic reviews, prospective cohorts, and recent mechanistic work using single-cell, spatial, organoid, multi-omics, microbiota, or regenerative-medicine approaches. The selection criteria excluded duplicate publications, studies unrelated to endometrial receptivity/RIF, non-reproductive tissues without direct mechanistic relevance, and records lacking sufficient bibliographic information. The final revised reference list contains 142 publications.

## 2 Clinical assessment: actionable uterine factors before empirical add-ons

Clinical assessment of RIF and other assisted reproductive technology (ART) failures should be grounded in a systematic and clinically meaningful diagnostic pathway, rather than moving directly to empirical endometrial treatment. Endometrial testing has evolved from conventional histologic “dating” toward molecular timing, assessment of proliferative and differentiation functions, and screening for inflammatory markers. Regardless of technological advances, however, its core objective should be to identify abnormalities that change clinical decision-making, not merely to add more tests (4). Accordingly, evaluation of suspected endometrial factors should still include basic pathology and assessment of intrauterine structure, including correctable abnormalities such as endometrial polyps, hyperplastic lesions, inflammatory changes, and retained products of conception. At the same time, the initial evaluation of RIF should cover the continuum of gamete and embryo quality, laboratory culture conditions, the uterine environment, and embryo transfer timing. Only after these baseline factors have been adequately assessed should emerging areas such as chronic endometritis (CE), vaginal or endometrial microbiota abnormalities, immune phenotypes, and immunomodulatory therapy be discussed with patients on the basis of sufficient evidence-based information (10). This pathway helps avoid premature attribution of complex assisted reproduction failure to a single endometrial defect and may reduce add-on therapies without a clear etiologic rationale. The diagnostic and assessment strategies discussed in this section are summarized in **Table 2**.

Thin endometrium is one of the organic or functional abnormalities most frequently considered in clinical assessment. It is commonly defined as a mid-luteal endometrial thickness of no more than, or below, 7 mm, and is associated with lower implantation rates, clinical pregnancy rates, and adverse ART outcomes (11). Its etiology is clearly heterogeneous and may involve age-related declines in endometrial responsiveness, intrauterine factors, an inflammatory microenvironment, long-term oral contraceptive use, effects of ovarian stimulation drugs, or unexplained primary endometrial underdevelopment. Mechanistically, thin endometrium is not simply “insufficient thickness”; it may represent a composite of impaired angiogenesis, inadequate local perfusion, inflammatory activation, restricted stromal-cell proliferation, cellular senescence, collagen deposition, and abnormal immune-cell composition (12, 13). Current strategies intended to improve thin endometrium include hormonal therapy such as estrogen, vasoactive drugs, granulocyte colony-stimulating factor (G-CSF), platelet-rich plasma (PRP), and regenerative-medicine interventions. The mechanisms, suitable populations, and true efficacy of these approaches remain incompletely defined. Therefore, therapies for thin endometrium should be described as investigational or selectively applicable strategies requiring further validation, rather than as established standard treatments.

With advances in molecular diagnostics, assessment of endometrial receptivity has expanded from morphology and thickness to window of implantation (WOI) synchrony, immune activation, microbiota composition, noncoding RNAs, extracellular vesicles (EVs), glycans, and other emerging biomarkers. These indicators provide research directions, but clinical translation requires independent cohort validation and etiology-specific trials (14). Transcriptome-based receptivity timing tests such as the endometrial receptivity array (ERA) can classify WOI-related molecular states, yet systematic-review evidence in euploid embryo-transfer cycles has not shown consistent improvement in clinically meaningful reproductive outcomes (15). Routine ERA or transcriptome-based receptivity testing is therefore not recommended for unselected infertile patients or routinely for patients with RIF, including euploid embryo-transfer settings, because classification of WOI timing has not been shown to consistently improve reproductive outcomes. Use should be restricted to research settings or exceptional individualized circumstances. By contrast, discovery transcriptomic and multi-omics profiling can reveal candidate RIF-associated molecular signatures, including inflammation-related pathways such as MAPK and PI3K-Akt, but these signatures require external replication, prospective validation, and clinical-utility testing before guiding treatment (16).

Management of organic intrauterine and uterine lesions likewise requires a clear distinction between a finding that may affect receptivity and an intervention that has proven reproductive benefit. Endometrial polyps, adenomyosis, and fibroids may be accompanied by molecular-expression changes unfavorable to embryo implantation, but imaging alone is not equivalent to a surgical indication. For myomectomy and adenomyosis-related surgery, lesion location, cavity involvement, symptoms, previous reproductive history, and surgical risk should be considered individually (5). For polyps, evidence synthesis in subfertile women younger than 40 years suggests relatively clear reproductive benefit from hysteroscopic polypectomy before intrauterine insemination; evidence in IVF/ICSI remains more heterogeneous (17). Thus, targeted removal after a confirmed polyp should be distinguished from routine screening hysteroscopy.

## 3 Cellular and immune niches revealed by single-cell and spatial omics

Single-cell RNA sequencing and spatial omics are shifting endometrial receptivity research from bulk-tissue averages toward a niche model shaped by cell type, spatial region, and temporal state. Spatial transcriptomic datasets from mid-luteal RIF endometrium have shown that epithelial, stromal, vascular, and immune compartments differ by region and that bulk analysis can obscure local abnormalities (18, 19). Time-series single-cell mapping further indicates that receptivity depends on coordinated stromal decidualization, progressive epithelial transition, and balanced inflammatory status rather than on a single marker (20). For the purposes of clinical interpretation, these findings can be organized into four interacting modules: stromal decidualization, epithelial adhesion and polarity, immune-epithelial niche regulation, and repair failure in thin or aging endometrium. This spatiotemporal niche framework is illustrated in **Figure 1**.

**Figure 1:**
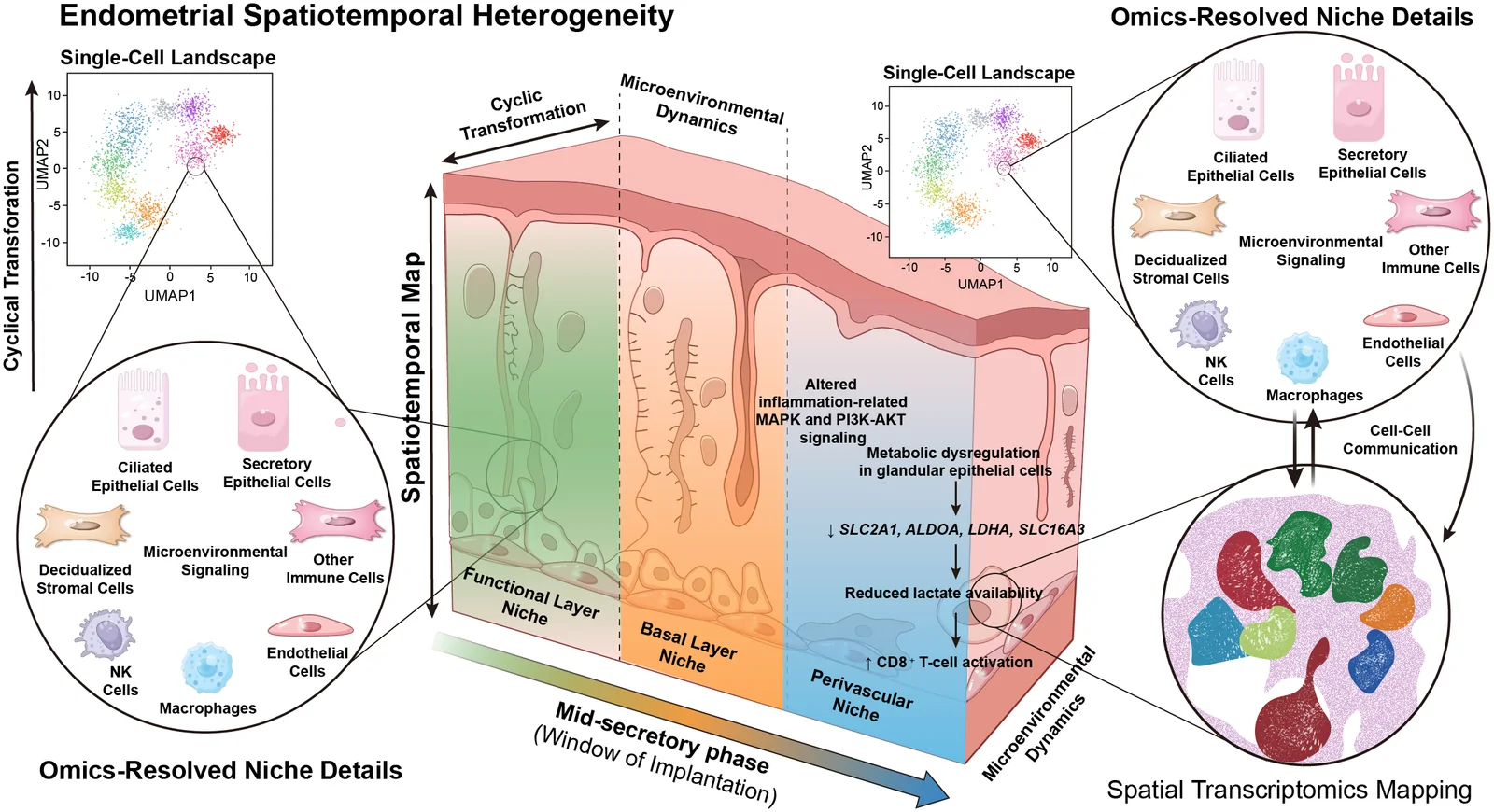
Spatiotemporal heterogeneity of the endometrial microenvironment during the window of implantation.

The first module is stromal decidualization. Studies implicate inflammatory signaling (21), hormone-response imbalance (22), cellular senescence (23), epigenetic regulation (24), cytoskeletal remodeling (25), and progesterone-related communication (26) in impaired stromal maturation. Rather than treating individual genes as independent diagnostic markers, these data suggest that stromal dysfunction reflects a coupled disturbance in decidual transformation, tissue architecture, inflammatory tone, and embryo–endometrial communication. Most supporting evidence remains mechanistic, preclinical, or based on small patient cohorts, so these pathways should be viewed as candidate mechanisms and possible targets for future validation rather than as established therapeutic entry points.

The second and third modules involve epithelial adhesion and polarity together with the immune-epithelial niche. Epithelial studies link altered polarity loss and trophoblast adhesion (27), interferon-related signaling (28), immune-cell-associated proteomic signatures (29), LIF-associated pathways (30), and glandular epithelial state (31) to implantation-related phenotypes. These findings suggest that epithelial competence and immune tolerance are interdependent rather than separate processes. They are useful for defining biological modules of receptivity, but their predictive value, reproducibility across cycle phases, and ability to guide treatment remain insufficiently established.

An additional inflammatory-perturbation study further illustrates how single-cell data should be interpreted within the evidence-to-translation framework. Lund and colleagues used an LPS-induced experimental model at the maternal-fetal interface and reported increased pro-inflammatory cytokine signals, altered proportions of immune cells, fibroblasts, endometrial stromal cells, and epithelial cells, and disrupted ligand-receptor communication involving embryo-maternal, stromal-epithelial, and stromal-immune pathways (32). This work is relevant because it links inflammatory exposure to cell-cell communication networks, but it remains an experimental inflammatory model. It should not be treated as direct evidence of a human RIF-specific mechanism or as evidence for a clinical treatment effect.

The fourth module is repair failure in thin, aging, or injured endometrium. Single-cell studies indicate that thin endometrium may combine impaired stromal proliferation, epithelial and stromal senescence, abnormal vascular and perivascular remodeling, altered macrophage or NK-cell abundance, and disrupted cell-cell communication (12). Mechanistic literature adds insufficient perfusion, hypoxia-related signaling, chronic inflammation, and epigenetic change to this repair-failure model (13). Small RIF subgroup data suggest that thin-endometrium RIF and normal-thickness RIF may represent different biological patterns, but the available cohorts are too small to support stable classification (33). Aging-related work similarly cautions that senescence-like programs may be pathological in some contexts while also participating in physiological implantation (34). Repair-oriented interventions therefore need to demonstrate functional restoration, live-birth benefit, and reproductive safety, not only thicker endometrium or short-term pregnancy signals.

Overall, single-cell analysis, spatial omics, and three-dimensional models provide a more refined mechanistic framework for understanding RIF and endometrial-factor infertility, but most current findings remain at the stage of candidate biomarkers and functional validation. Earlier work has linked polyps, adenomyosis, Asherman syndrome, CE, and Mullerian anomalies to endometrial integrity, receptivity, and RIF, indicating that clinical phenotypes still need to be interpreted together with molecular mechanisms (35). Literature on receptivity biomarkers suggests that testing systems are expanding from imaging indicators such as endometrial thickness, morphology, blood flow, and peristalsis to multilayered indicators including pinopodes, integrin alpha-v beta-3, osteopontin, HOXA10, ERA, and the microbiome, but clinical application remains to be validated (36). Literature on biomolecular markers of RIF further suggests that hormone levels, angiogenesis, immune regulation, and genetic polymorphisms may all participate in RIF, but available studies are often small and results differ across populations, which is insufficient for establishing a stable diagnostic panel (37). Notably, an exploratory RNA-sequencing study of mid-luteal endometrium from 107 infertile women did not identify differentially expressed genes or cell-enrichment features that could reliably distinguish implantation success from failure; it only suggested that the nonpregnant group might have WOI displacement (38). For clinical and immunological interpretation, these findings are most useful when they define cell-specific hypotheses that can be tested across cycle phase, embryo status, disease subtype, and reproductive outcome. Future studies should therefore combine spatial omics, time-series single-cell analysis, endometrial organoids, stromal-epithelial-immune co-culture systems, and prospective clinical cohorts to determine whether candidate mechanisms are reproducible, actionable, and clinically translatable (39, 40). These spatiotemporal niche relationships are summarized in **Figure 1**.

The schematic integrates single-cell and spatial transcriptomic perspectives on mid-secretory endometrial remodeling. A receptive endometrium contains functionally distinct luminal epithelial, glandular epithelial, stromal, immune, endothelial, and perivascular niches. Cyclical transformation is accompanied by dynamic cell-cell communication, extracellular-matrix remodeling, metabolic adaptation, and signaling changes involving inflammatory, MAPK, and PI3K-AKT pathways.

## 4 Disease-modified immune-cellular receptivity: endometriosis, polycystic ovary syndrome, and metabolic context

Disease-related impairment of receptivity is best interpreted through a consistent sequence: disease-associated endometrial phenotype, stromal/epithelial/immune mechanism, single-cell or multi-omics evidence, clinical human evidence, uncertainty, and translational implication. To preserve mechanistic depth, this section focuses on endometriosis and PCOS as the two principal disease contexts, whereas obesity, systemic metabolic inflammation, and selected environmental or epigenetic factors are discussed only as contextual modifiers rather than as coequal disease modules. In endometriosis, infertility cannot be explained only by pelvic anatomy, ovarian reserve, or embryo quality; the eutopic endometrium may also show a nonreceptive immune-cellular state. Single-cell analysis of minimal or mild endometriosis reported weakened cyclic immune changes, enhanced proinflammatory signaling, and fewer secretory-phase epithelial cells, suggesting impaired immune tolerance and epithelial maturation during the receptive window (41). Human reviews and multi-omics studies further implicate progesterone resistance, estrogen predominance, oxidative stress, ferroptosis, abnormal glycosylation, extracellular-matrix remodeling, microbiota dysbiosis, and altered NK-cell or macrophage activity (42–45). These findings deepen the mechanism but remain largely associative, disease-contextual, or model-based; they do not justify routine empirical immunotherapy, antioxidant treatment, or unselected endometrial intervention.

The immune microenvironment in endometriosis also illustrates why disease labels cannot be converted directly into treatment algorithms. Reviews report abnormalities in macrophages, immature dendritic cells, regulatory T cells, and NK-cell activity, but sample size, menstrual phase, hormonal exposure, and assay platforms vary substantially (45, 46). Tissue studies suggest links among macrophages, uNK cells, plasma cells, VEGF-A, and abnormal angiogenesis, and organoid or machine-learning models can test hormone resistance, inflammation, and candidate molecules such as EHF (47–49). The translational boundary is that these platforms identify disease-modified receptivity phenotypes and trial-enrichment hypotheses, not validated diagnostic panels or therapeutic targets.

PCOS-associated receptivity impairment should likewise not be reduced to anovulation. Although anovulation is a major cause of PCOS-related infertility, pharmacologic correction of ovulation and transfer of high-quality embryos do not fully eliminate implantation and pregnancy-loss concerns. Mechanistically, abnormal endometrial hormone and receptor expression and altered receptivity-marker profiles may weaken epithelial adhesion, whereas insufficient energy supply, impaired perfusion, chronic low-grade inflammation, metabolic disturbance, and microbiota-related signals may alter the local immune-cellular environment (50–52). These observations support an interacting endocrine, epithelial, metabolic, and immune framework for persistent endometrial dysfunction despite correction of ovulatory abnormalities, but the relative contribution and causal direction of individual pathways remain incompletely resolved.

Recent experimental work provides a more specific immune-stromal mechanism. In PCOS-like mice and patient-derived endometrial organoids, suppression of the IL-22-STAT3-IGFBP5 axis was associated with impaired receptivity, whereas exogenous IL-22 or IGFBP5 restored implantation-related functions in experimental models (53). These findings support a candidate immune-stromal signaling pathway but remain preclinical and should not be interpreted as evidence that IL-22 or IGFBP5 therapy improves human reproductive outcomes. Integrated transcriptomic and machine-learning analysis have additionally identified shared candidate genes between PCOS and RIF, including GLIPR1 and MAMLD1, together with tricarboxylic-acid-cycle disturbance and immune-cell-activation signals (54). Because these findings derive primarily from public datasets, algorithmic selection, and limited RT-PCR validation, they remain exploratory candidate-biomarker evidence until reproduced in prospectively phenotyped human cohorts with clinically meaningful reproductive outcomes.

Obesity and systemic metabolic inflammation provide a shorter contextual modifier of this disease framework. Obesity may place the endometrium in an inflammatory, oxidative, insulin-resistant, adipokine-altered state during the WOI, but causal links among obesity, endometriosis, PCOS, embryo quality, and endometrial receptivity remain incompletely resolved (55, 56). GLP-1 receptor agonists may improve fertility indirectly through metabolic and ovulatory effects, yet direct endometrial effects and early-pregnancy safety require separate evaluation (57). Gut microbiota dysbiosis may participate in PCOS through insulin resistance, inflammation, and hormone metabolism, but no specific microbial pattern has been proven to cause human receptivity failure (58). Environmental endocrine disruptors and HOXA10/HOXA11 epigenetic findings are therefore treated here as contextual modifiers rather than coequal disease sections: they may identify modifiable exposure or molecular contexts, but they do not yet define ART treatment algorithms (59–62).

## 5 Immune dysregulation and chronic endometritis

The immune response at the embryo–endometrial interface is not simply “immune suppression.” Rather, it maintains a dynamic balance among embryo adhesion, stromal decidualization, vascular remodeling, trophoblast invasion, and resolution of inflammation. Uterine natural killer cells (uNK cells) are among the major lymphocyte populations in early pregnancy decidua, accounting for approximately 50%-70% of decidual lymphocytes. Their low-cytotoxic, proangiogenic, and tissue-remodeling phenotype helps support trophoblast invasion, embryonic development, and placentation. Under infection, persistent inflammation, or abnormal activation, however, NK cells may shift toward a more cytotoxic phenotype and participate in pathological processes such as preeclampsia, recurrent miscarriage, endometriosis, and RIF (63). This bidirectional feature indicates that peripheral NK-cell counts cannot substitute for evaluation of local decidual or endometrial NK-cell function. Systematic review and meta-analysis show that endometrial CD56+ uNK levels are higher on average in patients with RIF, but pooled heterogeneity is high, uNK levels are not consistently associated with pregnancy outcomes, and peripheral NK cells do not reliably correlate with uNK cells (64). Therefore, uNK abnormalities are better interpreted as signals of local immune-environment disturbance than as a single indicator for routine immunotherapy decisions. Dyslipidemia, PCOS, and metabolic inflammation may also participate in RIF or reduced receptivity by affecting NK-cell subsets, macrophage proportions, the endometrial transcriptome, and local microbiota composition, but these associations require validation using standardized assays and prospective cohorts (65, 66).

Beyond NK cells, T cells, macrophages, regulatory lymphocytes, and cytokine networks jointly form the immune quality-control system during implantation. Immunobiological research conceptualizes the implantation immune response as a “quality-control” process: when embryonic and endometrial conditions are appropriate, it promotes adhesion, decidualization, and immune tolerance; when the tissue environment is unfavorable, it limits receptivity and may prevent abnormal pregnancies from progressing (67). This concept helps explain why increased inflammation or immune activation does not necessarily indicate pathology. The key determinants are the timing, location, intensity, and resolution of the inflammatory response. Recent single-cell, *in vitro*, and mouse experiments further suggest that epithelial metabolic status may influence local immune balance through T-cell regulation. In unexplained RIF endometrium, glycolysis and lactate production are reduced; genes related to glucose uptake, metabolism, lactate production, and lactate export, including SLC2A1, ALDOA, LDHA, and SLC16A3, are downregulated; and CD8+ T-cell proliferation and activation are increased. Lactate supplementation in mice reversed part of the implantation impairment, while the effect of lactate inhibition on implantation was attenuated after CD8+ T-cell depletion, suggesting that a glandular epithelial metabolism-CD8+ T-cell axis may participate in RIF immune imbalance (68). Nevertheless, RIF-related immune abnormalities are highly heterogeneous. Th1/Th2 ratio, NK cells, macrophages, autoantibodies, and interleukin networks may all be altered, but no unified immune phenotype exists across patients (69–71). Therefore, although endometrial immune profiling is a candidate research direction for pregnancy prediction and etiologic stratification, differences in testing time, cell definitions, sample source, cycle phase, and outcome indicators currently limit the development of a generalizable diagnostic model (72).

Chronic endometritis is among the better-defined inflammatory phenotypes linked to impaired receptivity and RIF, but its diagnostic heterogeneity is clinically important (73–77). When CE evaluation is clinically indicated, endometrial biopsy with histopathologic plasma-cell assessment, commonly supported by CD138 immunohistochemistry, should serve as the core confirmatory approach; hysteroscopic and microbiological findings are complementary rather than interchangeable substitutes. Biopsy timing, staining protocols, CD138 thresholds, and pathogen-detection strategies differ across studies, which helps explain why reported CE prevalence and treatment effects vary. Selective CE evaluation can therefore be discussed in patients with persistent RIF, recurrent pregnancy loss, or clinical features suggesting endometrial inflammation, but routine universal screening remains insufficiently supported. Antibiotic treatment should be reserved for a confirmed infectious or inflammatory phenotype and interpreted through antimicrobial stewardship rather than applied empirically.

Microbiota research further extends understanding of endometrial inflammation beyond CE, but a single microbial feature still cannot be interpreted directly as a receptivity defect. A prospective study in women with unexplained infertility found that nonpregnant patients had higher endometrial microbiota diversity, a lower proportion of Lactobacillus, and enrichment of potential pathogens such as Prevotella and Corynebacterium. *In vitro* exposure to butyrate induced inflammatory responses and weakened the epithelial barrier, but also upregulated some receptivity and decidualization markers, indicating that a change in a single microbial metabolite cannot be equated simply with functional improvement or impairment (78). A retrospective study of 271 endometrial biopsies found a CE prevalence of 7.4%, with no significant difference between patients with and without previous implantation failure. CE was also not associated with BCL6 overexpression or a nonreceptive ERA result, suggesting that CE does not stably overlap with other endometrial-test abnormalities (79). Female genital tract tuberculosis can also cause immune dysregulation and fertility impairment, but its etiology, tissue-destruction pattern, and treatment pathway differ from those of general CE or RIF, and the conditions should not be conflated (80). Overall, endometritis-related studies indicate that inflammation, microbiota, vasculature, and uterine dynamics may all participate in implantation failure, but differences in definitions, assays, and outcomes still prevent formation of a unified clinical pathway (81).

Macrophages and cytokine networks are important links among chronic inflammation, tissue repair, and abnormal receptivity. Macrophages participate in decidualization, vascular remodeling, embryo adhesion, and multiple reproductive diseases. Their M1/M2 polarization state may influence endometriosis-associated infertility, RIF, and CE, but different polarization markers, spatial distributions, and interaction networks with NK cells, T cells, and stromal cells require further clarification (82, 83). A machine-learning study based on data from 218 participants and validated with 40 clinical samples constructed a macrophage-endometrium interaction model with high AUCs in training and validation datasets. However, strong algorithmic performance does not equal real-world diagnostic effectiveness, and independent prospective populations are still needed for testing (84). Patient samples, *in vitro* cells, and a mouse CE model suggest that HMGB1 can induce macrophage pyroptosis, providing mechanistic evidence for inflammatory cell death in endometrial injury. Experimental inhibition results, however, cannot be directly extrapolated to therapeutic benefit in humans (85). Similarly, literature on immunotherapy lists tacrolimus, intravenous immunoglobulin, prednisone, intralipid, vitamin E, endometrial scratching, human chorionic gonadotropin (hCG), and other empirical approaches, which itself reflects the highly empirical nature of current treatment. Before validated immune diagnostic tools, standardized stratification, and adequate randomized trials are available, immune interventions should be restricted to clear indications and rigorous risk assessment (86–88). Taken together, immune mechanisms can guide hypothesis generation and trial enrichment, but immune-targeted therapy should remain phenotype-driven and outcome-tested rather than empirically applied. The contrast between balanced immune receptivity and CE-associated dysregulation is summarized in **Figure 2**.

**Figure 2:**
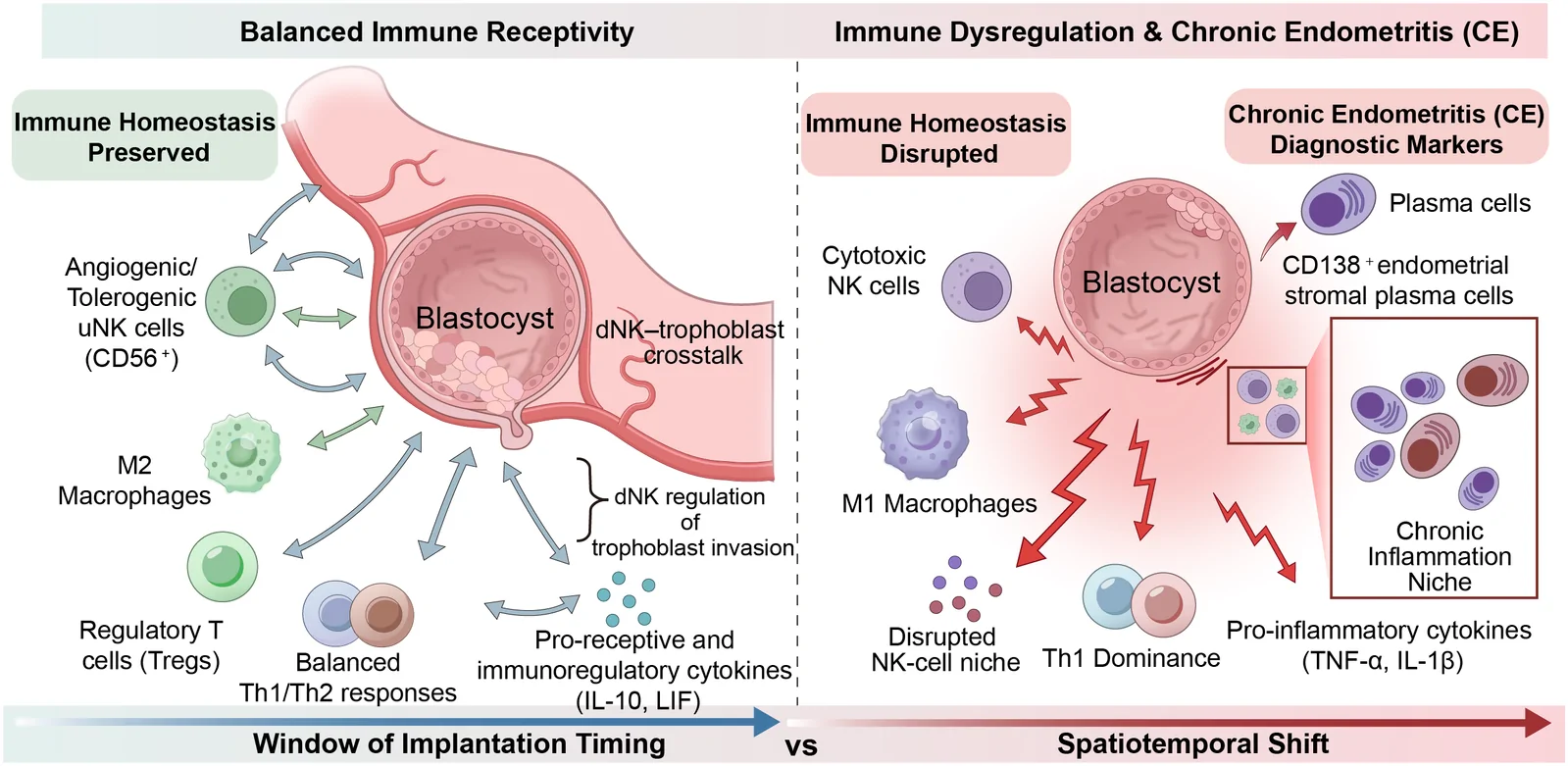
Immune balance and dysregulation in endometrial receptivity and chronic endometritis.

The schematic contrasts a physiologically receptive immune microenvironment with inflammatory endometrial states associated with CE. Balanced receptivity is characterized by tolerogenic uterine natural killer cells, M2-like macrophage features, regulatory T cells, anti-inflammatory cytokine signaling, and coordinated decidual immune-cell interactions. Reported immune dysregulation includes increased cytotoxic uterine natural killer-cell activity, M1-like macrophage polarization, Th1-skewed inflammation, pro-inflammatory cytokine production, plasma-cell infiltration, and CD138-positive staining. These features are context-dependent mechanisms and diagnostic clues, not stand-alone indications for empirical immunotherapy.

## 6 Extracellular communication, metabolism, and embryo–endometrium crosstalk

Extracellular vesicles, especially exosomes, provide a mechanistic perspective on gamete development, embryo quality, receptivity, and early embryo-endometrium communication. Exosomes released by reproductive tissues carry proteins, lipids, miRNAs, mRNAs, and other noncoding RNAs and may influence oocyte development, ovarian aging, PCOS, uterine disease, tubal function, immune regulation, and endometrial repair (89). In ART, exosomes are proposed to participate in embryo-endometrium crosstalk, embryo culture, cryopreservation, endometrial repair, and ovarian recovery. However, vesicle isolation, purification, characterization, sample source, assay platform, and functional-validation workflows remain insufficiently standardized; exosomes therefore cannot yet be regarded as mature diagnostic or therapeutic tools (90).

During implantation, exosomes derived from the embryo, semen, and uterine fluid may carry nucleic acids, proteins, and other signaling molecules that participate in epithelial adhesion, stromal decidualization, immune regulation, and embryo–endometrial recognition (91). These studies suggest that EVs may become noninvasive sources for evaluating oocyte quality, semen function, embryo viability, and endometrial status. Literature on EV-based diagnostics has summarized candidate markers such as follicular-fluid EV-miRNAs, seminal exosomal proteins, embryo-secreted vesicles, and uterine-lavage markers, but it also clearly states that these assays require validation studies and further development of technical platforms before they can enter routine ART diagnostic workflows (92). In human endometrial immunohistochemical studies, women with unexplained infertility and fertile controls show cycle-dependent differences in CD63 and CD9 expression. CD63 is higher in the proliferative-phase luminal and glandular epithelium of infertile women, whereas CD9 is elevated in secretory-phase infertile endometrium, suggesting that exosome-related markers may reflect endometrial status (93). However, such findings remain candidate-biomarker discoveries and are substantially influenced by menstrual cycle phase, sampling time, tissue region, and assay thresholds; they cannot yet be used alone to determine receptivity or guide treatment.

Lipid metabolism provides another important mechanistic clue for understanding endometrial receptivity. Lipids are not only energy sources and structural components of membranes but also inflammatory, vascular, hormonal, and cell-communication signals involved in embryo implantation, endometrial decidualization, and embryo–endometrial interface remodeling. Literature on lipid metabolism places fatty acids, prostaglandins, endocannabinoids, lysophosphatidic acid, sphingosine-1-phosphate, and cholesterol-derived steroid hormones within regulatory networks for implantation and decidualization. It suggests that fatty acid-related proinflammatory responses at the embryo–endometrial interface may promote pregnancy establishment through prostaglandin signaling, whereas endocannabinoids, lysophosphatidic acid, and sphingosine-1-phosphate are related to endometrial receptivity, embryo positioning, and decidualization. In addition, changes in serum cholesterol across the menstrual cycle suggest a steroidogenic requirement during the WOI, and the lipidome of endometrial fluid may provide a new direction for noninvasive assessment of receptivity (94). At present, however, lipidomics is mainly used for mechanistic analysis and candidate-biomarker screening and is not established for routine clinical assessment of endometrial receptivity.

Semen exposure is another form of extracellular communication linking male factors with peri-implantation endometrial status. Seminal fluid can influence uterine immune and vascular transcriptional states around ovulation, and human and mouse RNA-sequencing studies support effects on the endometrial transcriptional environment (95). This direction shows that receptivity is not determined solely by female endocrine and local endometrial factors. However, human pathways, individual variability, ART relevance, and clinical utility remain uncertain.

Psychological stress and diet are retained only as systemic modifiers. Stress has been associated with functional endometrial abnormalities, RIF, recurrent miscarriage, and other adverse reproductive outcomes, but it should not be treated as a specific endometrial etiology (96). Anti-inflammatory dietary patterns such as the Mediterranean diet may be associated with better natural fertility, ART outcomes, or semen quality through lower inflammation, improved lipid composition, and reduced oxidative stress, but causal pathways and intervention adherence remain heterogeneous (97). These factors are therefore best framed as supportive health contexts rather than substitutes for etiologic diagnosis or core ART treatment.

Overall, EVs, lipid metabolism, semen exposure, stress, and diet help map local and systemic communication at the embryo-endometrium interface, but the section is intentionally concise to preserve the main focus on immune-cellular receptivity niches. These factors remain mechanistic, associative, or supportive unless prospective studies show reproducible effects on ongoing pregnancy, live birth, and safety.

## 7 Microbiota-immune interactions: association, sampling, mechanisms, and translation

The female reproductive tract microbiota may influence endometrial receptivity and ART outcomes through local immunity, endocrine metabolism, and mucosal barrier function, but current evidence remains dominated by associative studies and methodological exploration. In general, Lactobacillus-dominant communities, especially those dominated by Lactobacillus crispatus, are usually considered associated with low inflammation, low pH, and better conception or embryo implantation outcomes. In contrast, reproductive tract or gut dysbiosis may indirectly affect ovulation, embryo quality, and endometrial receptivity through proinflammatory responses, abnormal hormone metabolism, epithelial barrier impairment, and metabolic-inflammatory states related to PCOS or endometriosis (98). This view is consistent with the overall trend in the literature on female reproductive tract microbiota that low microbial diversity and Lactobacillus dominance are more likely to be associated with better ART outcomes, but it does not mean that Lactobacillus dominance can be directly equated with a healthy endometrium or clinical benefit (99). Existing studies of the endometrial microbiota also emphasize that endometrial microecology may be influenced by age, hormonal fluctuation, immune responses, and CE and may be associated with implantation and infertility, but no consensus definition of a healthy composition has been established (100). Therefore, the primary task of reproductive tract microbiota research is not simply to identify “beneficial” or “harmful” bacteria, but to establish unified standards for sampling, extraction, sequencing, culture, negative controls, bioinformatic analysis, and clinical-outcome assessment.

Sampling and contamination control are central bottlenecks in translating endometrial microecology research. The uterine cavity is a low-biomass environment, and samples are highly susceptible to contamination from transvaginal sampling, the cervical canal, reagent background DNA, and laboratory workflows. Therefore, even when some studies observe Lactobacillus dominance within the uterus, it remains difficult to fully exclude contamination from vaginal sources (101). Literature on sampling techniques further indicates that there is no accepted healthy endometrial microbial profile and no unified method for endometrial microbiota sampling, directly limiting reproducibility and comparability across cohorts (102). A study using double-lumen embryo-transfer catheters to collect endometrial fluid from 53 women undergoing IVF found that only 8% of cases showed endometrial Lactobacillus dominance, that endometrial and vaginal taxonomic compositions differed substantially, and that no single bacterium was directly associated with pregnancy success. Shannon evenness in the endometrium was actually higher among women who became pregnant, indicating that the model in which Lactobacillus dominance is necessarily favorable is overly simplified (103). Conversely, a paired study of 120 women undergoing frozen-thawed embryo transfer showed that the nonpregnant group had a lower proportion of vaginal Lactobacillus and a higher proportion of Gardnerella, whereas uterine-cavity diversity did not differ significantly and only some genera changed in abundance. These findings suggest that the vaginal microbiota, because of its higher biomass and more stable sampling, may more readily provide reproducible predictive signals than low-biomass uterine-cavity samples (104).

Whether the endometrial microbiota is independent of the vaginal microbiota remains unresolved. A study of 71 paired vaginal and endometrial samples found that most samples from both sites were Lactobacillus dominant and that the most abundant Lactobacillus species within the same patient were often shared between sites. However, endometrial samples usually had higher diversity, and the choice of 16S rRNA variable region affected dysbiosis classification and taxonomic results (105). Culturomics provides complementary evidence. In paired cultures from 10 subfertile women, only an average of 28% of species overlapped between vagina and endometrium within the same patient, and some species were found only in the endometrium, suggesting that the endometrial microbiota may not be a simple copy of vaginal contamination. However, because clinical samples still need to pass through the vaginal-cervical route, cross-contamination cannot be fully excluded (106). Another proof-of-concept culturomics study also showed that high-throughput culture could identify multiple bacterial and fungal species in low-biomass endometrial samples, including many species that had not been fully described in previous sequencing studies, indicating that culturomics can serve as an important complement to sequencing (107). Future endometrial microecology research should therefore not rely on 16S sequencing alone. Instead, it should integrate culturomics, metagenomics, metabolomics, negative controls, and host-response indicators to distinguish viable colonization, transient carriage, contamination signals, and host responses induced by microbial residues.

Studies using microbiota to predict embryo implantation and ART outcomes provide candidate directions for individualized assessment, but model performance is not equivalent to clinical readiness. A 16S and metagenomic study of 239 infertile patients and 17 healthy women found that reproductive tract microbiota composition could distinguish implantation success from repeated failure and established a predictive model with a training AUC of 0.913 and a validation AUC of 0.784. Functional analysis suggested that women with repeated failure had higher vaginal pH, lower Lactobacillus abundance, and enrichment of the L-lysine synthesis pathway (108). Another vaginal 16S study including 194 infertile women and 100 healthy controls showed differences in microbial diversity and the abundance of several genera in infertile and RIF-related populations and constructed a machine-learning model. However, its metabolic pathways were mainly inferred from PICRUSt and still require validation by metagenomics, metabolomics, and prospective clinical outcomes (109). In addition, a study of 134 asymptomatic infertile patients found that patients with CE or polyps had different vaginal, cervical, and uterine-cavity microbiota in species composition and relative abundance compared with controls, but these differences mainly support disease association and do not prove that microbiota changes directly cause implantation failure (110). Microbiota prediction models are therefore currently better suited for research stratification than for direct decisions about antibiotics, probiotics, or embryo-transfer strategies.

The relationship between CE and the endometrial microbiota further illustrates the evidence pattern of relatively clear association but insufficient causality. CE may present with plasma-cell infiltration, abnormal immune-cell subsets, local cytokine changes, and impaired decidualization, and it may reduce receptivity by altering the endometrial immune microenvironment. However, its diagnosis, etiology, and treatment remain controversial (111). A prospective RIF study showed that patients with CE had higher endometrial microbiota diversity and evenness, enrichment of taxa such as Proteobacteria, and a greater tendency for Lactobacillus to be associated with non-CE status. Predicted functional pathways involved vitamins, cofactors, secondary metabolites, and the immune system and were associated with adverse reproductive outcomes (112). However, a study of 100 women who conceived naturally and chose pregnancy termination found that the overall endometrial microbiota was similar between CE and non-CE groups, with only some differences in genus abundance and interaction patterns. This indicates that CE compatible with natural conception does not correspond to a fixed microbial pattern (113). These findings suggest that CE is more likely a group of tissue inflammatory phenotypes than a single disease defined by one specific pathogen or fixed microbial combination. When applying CE microbiota findings clinically, pathology thresholds, sampling time, antibiotic history, intrauterine procedures, vaginal-cervical microbiota background, and subsequent reproductive outcomes should all be considered; otherwise, the diagnostic or therapeutic value of microbiota testing may be overestimated (114).

Translational microbiota research needs to move beyond whether bacteria are present toward whether microbial signals affect host function. The endometrial microbiota may interact with immune tolerance during the peri-implantation period, but low biomass, contamination risk, lack of a defined healthy core microbiota, and insufficient standardization remain inherent limitations (115). A recent review further emphasized that endometrial microbiota, cytokines, and growth factors may coordinate maternal immune adaptation and embryo implantation, but much of this synthesis includes broad mammalian and mechanistic evidence and should not be read as proof that microbiota supplementation improves human ART outcomes (116). Research on bacterial EVs similarly proposes a route by which microbial products could influence reproductive tissues, yet host-derived and bacterial vesicles, detection sensitivity, contamination control, and functional validation remain unresolved (117). At present, microbiota-targeted intervention remains investigational; altered taxa, predicted pathways, or short-term pregnancy signals are insufficient to justify routine antibiotics, probiotics, or transfer decisions (118, 119).

## 8 Reparative and regenerative ART add-ons: translational boundaries and clinical readiness

Growth factor-related interventions reflect a shift in assisted reproduction from focusing solely on embryo quality toward bidirectional embryo-endometrium regulation. Growth hormone (GH) and its downstream insulin-like growth factor-1 (IGF-1) signaling are among the earlier add-on interventions to receive attention. Earlier studies often attributed the potential benefit of GH to improvements in follicular development, granulosa-cell gonadotropin responsiveness, oocyte maturation, and embryo quality. More recent work suggests that GH and its functional receptor are also expressed in uterine and endometrial tissues and may participate in receptivity by promoting endometrial proliferation, regulating local hormonal responses, affecting implantation-related signaling, and influencing embryo implantation (120, 121). In RIF, thin endometrium, and older normal responders, GH has been proposed to have possible auxiliary effects beyond the ovary, and reduced uterine implantation sites after targeted deletion of the GH receptor in animal experiments provide mechanistic clues for an endometrial role. However, the clinical effect of GH supplementation during ovarian stimulation remains controversial. Studies differ in population selection, dose, timing, combination protocols, and outcome indicators. GH is therefore best described as an investigational adjunctive strategy with possible endometrial regulatory effects, rather than as an established treatment for RIF or thin endometrium. Any claimed benefit should be evaluated using ongoing pregnancy, live birth, perinatal safety, and cost-effectiveness, not only intermediate endocrine, ovarian, or endometrial markers.

PRP, G-CSF, hCG, peripheral blood mononuclear cells (PBMCs), and other intrauterine perfusion strategies are widely used to improve outcomes in thin endometrium or RIF, but their shared problem is that mechanistic plausibility is stronger than clinical certainty. Literature on refractory thin endometrium lists PRP, G-CSF, GH, stem cells, and their exosomes as major regenerative or pro-repair strategies and notes that these interventions can be applied through intrauterine perfusion, subendometrial injection, or systemic administration. Existing studies often use endometrial thickness, clinical pregnancy rate, or implantation rate as primary endpoints, whereas live birth rate, perinatal safety, and long-term reproductive outcomes receive insufficient attention (122). Although PRP studies have reported some positive signals in thin endometrium, diminished ovarian reserve, and RIF, preparation procedures, platelet concentration, leukocyte content, activation methods, number of instillations, treatment timing, and patient selection are not standardized. PRP should therefore still be regarded as an investigational strategy until large RCTs demonstrate live-birth benefit and acceptable safety (123–125). A Cochrane review including 12 randomized trials and 1,069 women concluded that the certainty of evidence for almost all outcomes was very low, and that the effects of intrauterine or ovarian PRP on live birth, pregnancy, miscarriage, and safety remain uncertain (126). Furthermore, the final PRP product may not be simply a platelet concentrate; it may contain cytokines and growth factors derived from platelets and peripheral blood mononuclear cells. Defining the composition, concentration, source, and biological effects of blood products is therefore a prerequisite for improving reproducibility (127, 128). Increases in endometrial thickness, implantation rate, or clinical pregnancy rate should therefore not be interpreted as improved receptivity or live-birth benefit unless confirmed by adequately powered trials. The major regenerative and intrauterine add-on strategies are summarized in **Figure 3** and **Table 3**.

**Figure 3:**
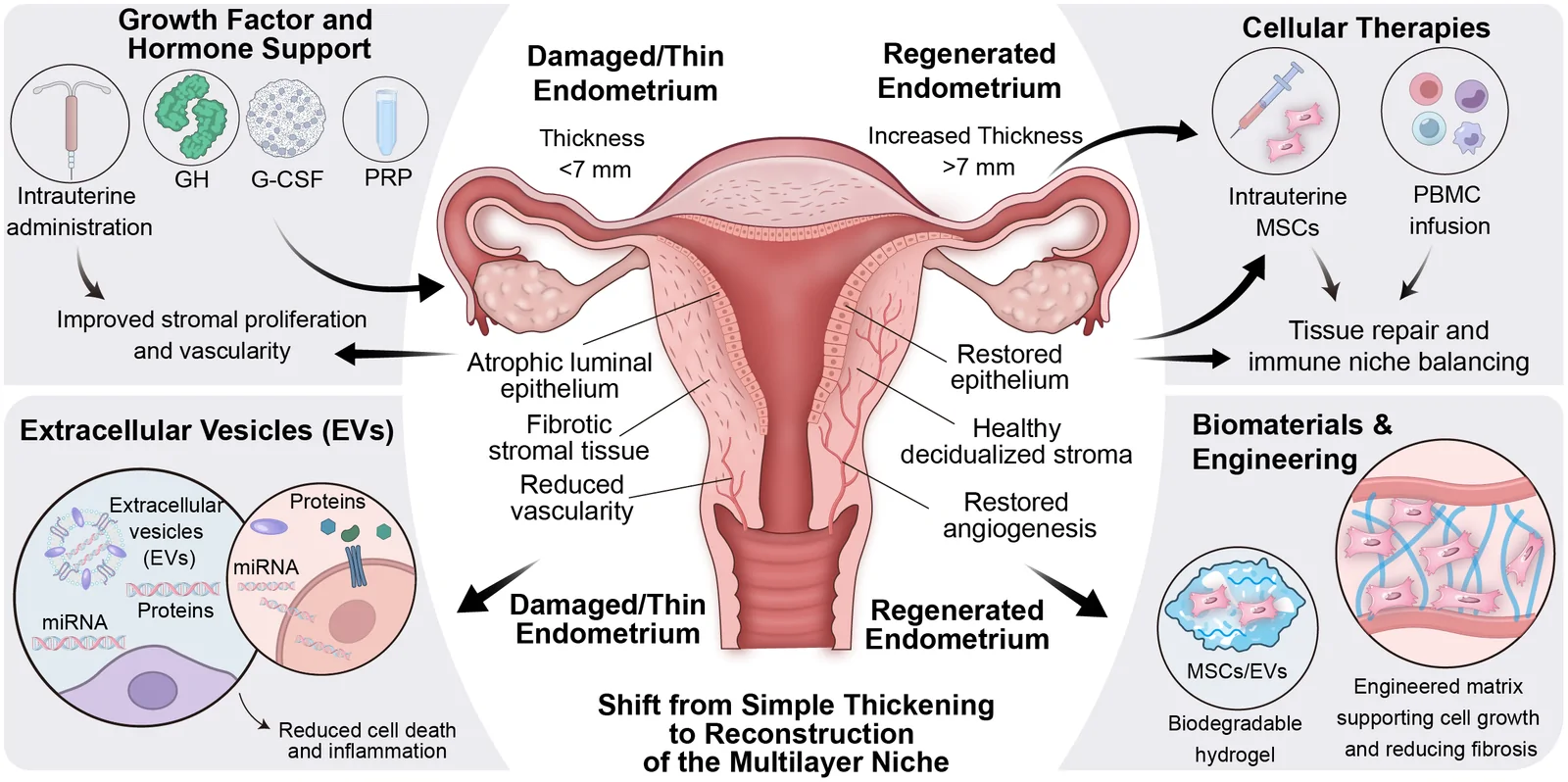
Mechanistic regeneration map for damaged or thin endometrium.

Other intrauterine perfusion and immunomodulatory approaches similarly require a clear boundary between possible biological activity and adequate clinical evidence. A 5-year observational cohort suggested that intrauterine G-CSF may increase embryo implantation rates in some patients with RIF, but other reproductive outcomes were not stable and endometrial-thickening data were incompletely recorded (129). A network meta-analysis ranked hCG, PBMCs, G-CSF, and PRP among higher-ranked intrauterine perfusion strategies, but it combined randomized trials, nonrandomized controlled studies, and prospective cohorts and had limited direct head-to-head comparisons (130). Literature evaluating endometrial ART add-ons, including immunotherapy, endometrial scratching, ERA, vasodilators, and hCG perfusion, concluded that robust evidence for clear efficacy and safety is lacking (131). Intrauterine perfusion should therefore be positioned as investigational or selectively considered, not as a routine standard process for RIF or thin endometrium.

Endometrial scratching deserves separate consideration because it has been widely marketed as an inexpensive implantation add-on. The biological rationale is that controlled endometrial injury might trigger repair-associated inflammatory signaling, cytokine release, and tissue remodeling favorable to implantation. However, a large pragmatic randomized trial found no live-birth advantage for endometrial scratching before IVF, including among women with previous implantation failure, and reported procedure-related pain and a small number of adverse reactions (132). The 2026 Cochrane update, including 24 studies and 7,234 women, concluded that the effect of endometrial injury on live birth and clinical pregnancy remains unclear when higher-quality evidence is prioritized, and that the procedure commonly causes mild to moderate pain and minor bleeding (133). Routine endometrial scratching should therefore not be recommended as an empirical ART add-on.

Stem cells, exosomes, and biomaterial delivery systems represent new directions in the repair of thin or scarred endometrium. Their core concept has shifted from simply increasing endometrial thickness to rebuilding the damaged endometrial microenvironment. Mesenchymal stem cells can migrate to sites of injury and regulate angiogenesis, cell migration, inflammatory responses, and tissue remodeling by secreting cytokines, chemokines, growth factors, proteins, and EVs. Current evidence, however, comes mainly from rodent models and limited human trials, and the relative contributions of cell differentiation and paracrine repair require further distinction (134). Materials-science studies show that adipose-derived stem-cell exosome hydrogels can promote endothelial-cell proliferation and tube formation *in vitro* and, in animal models of thin endometrium, increase angiogenesis, reduce fibrosis, and restore fertility. Dopamine-modified hyaluronic-acid artificial mucus carrying phytoestrogens and urine-derived exosomes can also improve endometrial thickness, receptivity, and fertility outcomes in rat models, potentially involving ER-NLRP3-IL1beta and Wnt-beta-catenin-TGFbeta-smad-related signaling (135, 136). These results, however, are mainly derived from biomaterial platforms and animal experiments and cannot be directly inferred as human efficacy. Translation still needs to address autologous versus allogeneic cell source, EV/exosome characterization, biomaterial carrier safety, local retention time, dose control, immune responses, batch-to-batch consistency, placentation, fetal development, perinatal outcomes, and long-term pregnancy safety.

Microecological modulation and nutritional-immunologic interventions have also been incorporated into some adjunctive treatment frameworks, but they should not be conflated with regenerative medicine. A randomized clinical trial of probiotics combined with vitamin D in women with RIF and thin endometrium showed a higher pregnancy rate, accompanied by improvement in the Th1/Th2 ratio, NK-cell activity, and cytokine profile. However, the primary endpoint was pregnancy rate rather than live birth rate, and the intervention was a combined regimen; it therefore cannot separately prove that probiotics or vitamin D can reliably improve ART outcomes by improving endometrial microecology (119, 137). Overall, growth factors, intrauterine perfusion, blood products, stem-cell exosomes, and biomaterials reflect a therapeutic shift from “improving endometrial thickness” to “reconstructing the endometrial niche.” Clinical translation must nevertheless be based on standardized preparation, clear indications, randomized controls, live-birth endpoints, perinatal safety, long-term reproductive safety, and cost-effectiveness. Improvements in thickness, candidate markers, implantation rate, or short-term clinical pregnancy rate should not be interpreted as proven clinical benefit. **Figure 3** maps the proposed biological repair mechanisms, whereas **Table 3** separately summarizes the level of clinical evidence, readiness, limitations, and current clinical position of these interventions. The translational map of reparative and regenerative add-ons is summarized in **Figure 3**.

The schematic separates repair mechanisms from clinical readiness by showing how hormonal support, growth factors, G-CSF, PRP, EVs/exosomes, mesenchymal stem cell-based approaches, PBMC infusion, and biomaterial-assisted delivery are hypothesized to influence stromal proliferation, angiogenesis, epithelial repair, immune-niche rebalancing, inflammation reduction, and fibrosis attenuation. The figure should be interpreted as a mechanistic map rather than evidence that these interventions improve live birth.

## 9 Discussion

The revised synthesis supports three conclusions. First, receptivity is dynamic, multicellular, and spatially organized rather than a single molecular state. Stromal decidualization, epithelial maturation, vascular remodeling, immune-cell balance, metabolic signaling, and microbiota-associated host responses are interdependent components of the peri-implantation niche. Second, RIF is heterogeneous and can be overdiagnosed if embryo probability, euploidy, transfer number, uterine anatomy, and disease context are not considered. Third, single-cell, spatial, organoid, and multi-omics approaches provide valuable mechanistic resolution, including time-series endometrial mapping and disease-overlap analysis, but they most often generate discovery evidence, candidate biomarkers, or preclinical support rather than clinically actionable tests (20, 138).

The weakest evidence lies at the point where prediction is assumed to equal utility. A transcriptomic timing classification, a high-AUC model, a microbial association, an immune-cell shift, or a regenerative response in animals may be biologically informative without showing that patient management improves. This distinction is particularly important for ERA-like receptivity testing, immune profiling, microbiota testing, EV or metabolomic biomarkers, and machine-learning signatures. These tools may support research stratification and hypothesis testing, but they should not be promoted as routine clinical decision tools until prospective validation demonstrates added value beyond existing assessment and improves ongoing pregnancy, live birth, or safety.

Current clinical practice should therefore avoid empirical escalation. Routine ERA or transcriptome-based receptivity timing tests are not recommended for unselected infertile patients or routine RIF evaluation. Peripheral or endometrial immune profiling should not be used as a stand-alone indication for immunotherapy. Routine endometrial scratching lacks robust live-birth benefit and adds discomfort or minor procedural risk. Indiscriminate microbiota testing, antibiotics, probiotics, PRP, G-CSF, PBMC infusion, stem cells, exosomes, and biomaterial-based repair should be treated as investigational or selectively considered according to a defined phenotype, not as general ART add-ons.

Future studies should be designed around the specific translation gap they intend to close. Mechanistic studies need cycle-phase control, spatial or cell-specific validation, perturbation experiments, and external replication. Diagnostic studies need harmonized RIF phenotyping, embryo/euploidy control where relevant, prespecified thresholds, independent validation cohorts, and comparison with existing clinical pathways. Interventional trials need clear indications, standardized protocols, adequate controls, live birth as a key endpoint, perinatal and long-term safety follow-up, and cost-effectiveness assessment. Only this sequence can distinguish a plausible mechanism from a clinically useful strategy.

In conclusion, immune-cellular, microecological, metabolic, extracellular, and regenerative research has substantially improved the biological map of endometrial receptivity failure. Its current clinical value is greatest when it refines phenotypes and trial design rather than when it encourages empirical add-ons. The field should move from accumulating candidate signals toward validating which abnormalities are reproducible, causally relevant, safely targetable, and able to improve live birth and reproductive safety.
